# Genomic and structural investigation on dolphin morbillivirus (DMV) in Mediterranean fin whales (*Balaenoptera physalus*)

**DOI:** 10.1038/srep41554

**Published:** 2017-01-30

**Authors:** Giorgia Beffagna, Cinzia Centelleghe, Giovanni Franzo, Giovanni Di Guardo, Sandro Mazzariol

**Affiliations:** 1 Department of Comparative Biomedicine and Food Science, University of Padua, Padua, Italy; 2 Department of Animal Medicine, Production and Health, University of Padua, Padua, Italy; 3 University of Teramo, Faculty of Veterinary Medicine, Teramo, Italy

## Abstract

Dolphin morbillivirus (DMV) has been deemed as one of the most relevant threats for fin whales (*Balaenoptera physalus*) being responsible for a mortality outbreak in the Mediterranean Sea in the last years. Knowledge of the complete viral genome is essential to understand any structural changes that could modify virus pathogenesis and viral tissue tropism. We report the complete DMV sequence of N, P/V/C, M, F and H genes identified from a fin whale and the comparison of primary to quaternary structure of proteins between this fin whale strain and some of those isolated during the 1990–‘92 and the 2006–‘08 epidemics. Some relevant substitutions were detected, particularly Asn52Ser located on F protein and Ile21Thr on N protein. Comparing mutations found in the fin whale DMV with those occurring in viral strains of other cetacean species, some of them were proven to be the result of diversifying selection, thus allowing to speculate on their role in host adaptation and on the way they could affect the interaction between the viral attachment and fusion with the target host cells.

Dolphin morbillivirus (DMV) was recently detected, by means of biomolecular analyses, in some species of cetaceans found stranded along the Italian coastline in 2013, such as striped dolphin (*Stenella coeruleoalba*) and bottlenose dolphin (*Tursiops truncatus*)^1^. In contrast with the two well documented striped dolphin mass mortality outbreaks in the Mediterranean Sea in 1990–1992 and 2006–2008, during the unusual mortality event occurred in 2013 along the Tyrrhenian coast of Italy, infected animals did not show typical Morbillivirus-related pathological findings. Although no definitive conclusions could be drawn, dolphin morbillivirus was deemed to be the most likely cause^1^. Furthermore, the recent report of a mortality cluster associated with DMV in Mediterranean fin whales (*Balaenoptera physalus*) stranded along the Italian coastline highlights the crucial importance of understanding the evolution of morbilliviruses^2^. Morbilliviral infection has been previously described in mysticetes from the Mediterranean basin^3^, as well as from the North Atlantic Ocean^4^ and the North Sea^5^. Nevertheless, no identification nor genomic and structural characterization studies of the viral agent have been hitherto carried out.

Similar to other Morbillivirus genus member, the acute (and often fatal) nature of the infection, the life-long immunity and the absence of latency of DMV suggest that large populations of susceptible hosts are necessary for viral maintenance and persistence in the marine environment^6^,^7^. However, this does not always seem to be the case, due to the limited number of individuals in such an extremely wide ecosystem. The ability to infect multiple species sharing the same environment could represent a strategy to circumvent this biological limit^7^,^8^. Unfortunately, the multi-host nature of DMV represents a particular threat to endangered populations, especially in those situations where they coexist with susceptible and apparently non-susceptible host species with hosts’ density leading to increased opportunity for infection^9^.

DMV is an unsegmented, linear negative-sense, single-stranded RNA virus displaying six different transcription units that encode six structural proteins and two virulence factor proteins^6^. RNA viruses are characterized by an extremely high mutation rate (i.e.~10^−2^–10^−5^ mutations/site/replication), which makes them extremely prone to genotypic and phenotypic changes leading to the emergence of variants with different immunological properties, virulence or host tropism^10^.

A range of cetacean host species are deemed to be susceptible to DMV infection, with some of them appearing to be less resistant^11^. In this respect, as reported by De Vries and coworkers (2015), the sensitivity of the different species is regulated by the interplay between the haemoagglutinin (H) viral antigen and the host’s SLAM/CD150 receptor, which also determines the species barrier. Bottlenose (*Tursiops truncatus*) and striped dolphins (*Stenella coeruleoalba*) are believed to be the most DMV susceptible species on the basis of the DMV-SLAM/CD150 affinity level, having also been the two species most severely involved in epidemic outbreaks worldwide^8^,^11^. Mortality clusters related to DMV have been occasionally described in a range of odontocetes, as in the case of long-finned pilot whales (*Globicephala melas*)^12^ and in mysticetes^2^. However, in the Mediterranean morbilliviral infection has been occasionally reported also in other orders rather than Delphinidae, often without any spatial relation with ongoing epidemic outbreaks, as in the peculiar case of the sperm whales’ (*Physeter microcephalus*) mass stranding occurred along the Central Adriatic coast of Italy in 2014^13^. Surprisingly, DMV has been also shown to jump into new hosts, as in the case of a cross-species infection in a captive harbor seal (*Phoca vitulina*)^14^. These observations appear to get along with those related to Canine Distemper Virus (CDV), which is considered to be the most promiscuous of all the known morbilliviruses, being able to infect different carnivore species and to cross the species barrier even into primates^15^.

Knowledge of the DMV genome is therefore essential to understand any structural changes that could modify viral infection’s pathogenesis and host tissue tropism^11^. The present study reports a genomic and structural characterization of DMV identified from a stranded fin whale. A comparison of primary to quaternary structure of proteins of DMV strains obtained from fin whale, striped dolphin and long-finned pilot whale was performed by means of a bioinformatic approach, in order to understand the role of each single aminoacidic variation.

## Results

### DMV genome study

DMV genome was detected in the brain, the lung, and the spleen of the newborn fin whale^2^. Sequences of cloned DMV genome fragments were analyzed. The conventional RT-PCR technique associated with viral cloning using plasmid vectors allowed the identification of the entire viral genome. The sequence of each single gene was deposited in Genbank as shown below: complete N gene (1573 bp), Acc. No. KU977449; complete P/V/C gene (1521 bp), Acc. No. KU977450; complete M gene (1008 bp), Acc. No. KU977451; complete F gene (1659 bp), Acc. No. KU977452; complete H gene (1814 bp), Acc. No. KU977453.

**Figure 1:**
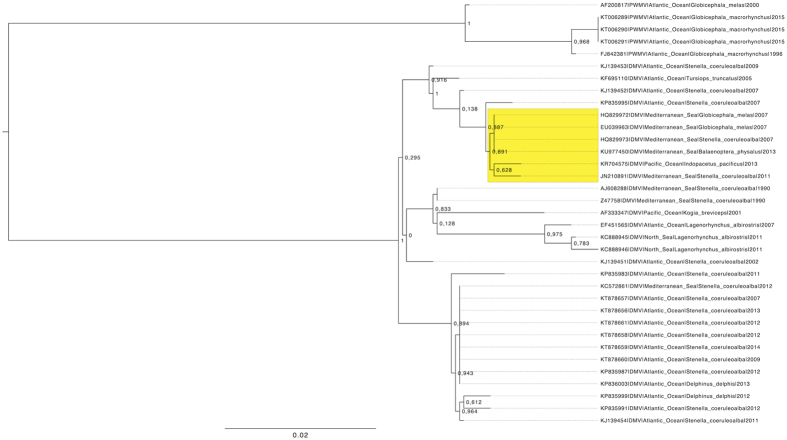
Maximum likelihood (ML) phylogenetic tree reconstructed on the basis of phosphoprotein (P) sequences of DMV and Pilot whale morbillivirus (PWMV) isolated over time in different seas. The bootstrap support is reported close to the corresponding node. The clade including the strain object of the current study is highlighted in yellow.

Afterwards, each sequence was compared to the DMV complete genome isolated from a striped dolphin involved in the 1990–’92 epidemic (Acc. No. AJ608288): these fragments showed a nucleotide percentage of identity between 99.10% and 99.80%. Phylogenetic analysis based on the partial phosphoprotein (P) gene demonstrated that the strain herein reported is closely related to a DMV strain recovered between 2007 and 2011, mainly in the Mediterranean Sea, during and after the 2006–2008 DMV outbreak (Fig. 1) (Genbank Acc. No. JN210891^16^, HQ829972^17^, HQ829973^17^, EU039963). Subsequently, the fin whale sequences were also compared to 2 closely related *Cetacean morbillivirus* (CeMV) isolates obtained during the 2006–‘08 outbreak from a striped dolphin (Genbank Acc. No. HQ829973) and a long-finned pilot whale (Genbank Acc. No. HQ829972); the percentage of identity was between 99.58% and 99.80% and between 99.52% and 99.90% respectively.

### Secondary structure prediction

PSIPRED was used in order to predict the secondary structure of gene N, gene M, gene H, gene F, and P/V/C gene, in agreement with what reported elsewhere (Genbank Acc. No. AJ608288, HQ829973 and HQ829972), as well as in order to investigate the corresponding sequences identified in our sample.

The observed secondary structures were the same for M and H gene: the secondary sequence structures of the two proteins showed a high degree of similarity in terms of α-helices and β-sheets in our sequences as in previously reported ones. On the contrary, the N, P/V/C and F gene-encoded proteins’ secondary structure showed differences between predicted sequences and ours.

**Figure 2:**
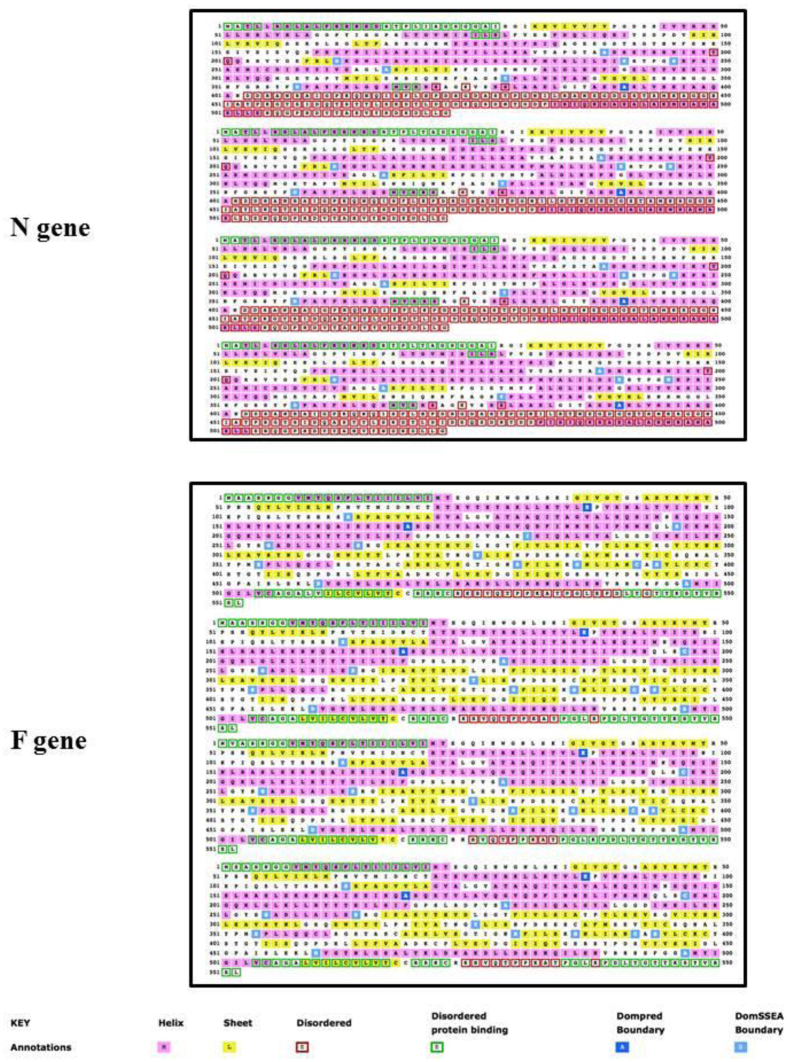
PSIPRED prediction of secondary structure changes’ maps. Top line: *Stenella coeruleoalba* strain (Genbank Acc. No. AJ608288); second line *Stenella coeruleoalba* strain (Genbank Acc. No. HQ829973); third line: *Globicephala melas* strain (Genbank Acc. No. HQ829972); bottom line: *Balaenoptera physalus* strain. N gene and F gene are depicted in box 1 and 2, respectively.

The N gene-encoded antigen in our fin whale showed a high degree of similarity in terms of α-helices and β-sheets to the AJ608288 reported F gene; HQ829973 and HQ829972 N gene showed more differences. In general, the N gene secondary structure differs between the sequences in terms of α-helices and changes that are expected to alter the protein function (Fig. 2). Moreover, the fin whale F protein showed differences, especially in the disposition of boundary aminoacids. The fin whale F gene is different from both AJ608288 and the 2 viral sequences isolated in the 2006–‘08 epidemic, in particular in the C-terminal part of the protein (Fig. 2).

The P/V/C gene product of our fin whale showed a greater amount of α-helices in comparison with what reported in the literature, in particular from residues 185 to 275 of the protein.

### Nucleotide, aminoacid sequence comparison and homology modelling

Differences in nucleotide and aminoacid pairwise raw distance between the fin whale’s and striped dolphin’s strain are reported in Table 1.

**Figure 3:**
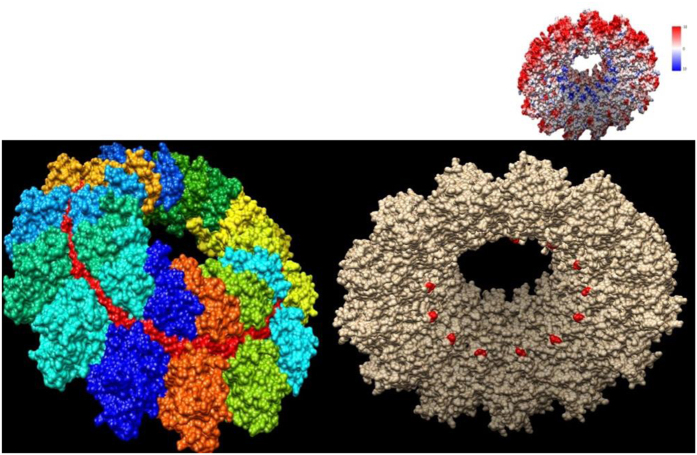
Structure of the nucleocapsid-RNA complex of the DMV strain detected in a *Balaenoptera physalus* predicted through homology modelling. In the top view (left) the different capsomers are shown by color coding, while the bound viral RNA is depicted in bright red. In the bottom view (right) the aminoacid position variations between the DMV isolates obtained from *Stenella coeruleoalba* and *Balaenoptera physalus* one are highlighted in red. This region is negatively charged and interacts with the positively charged, outside-top, one (top-right insert), which is located in the underlying layer of the nucleocapsid helix.

**Figure 4:**
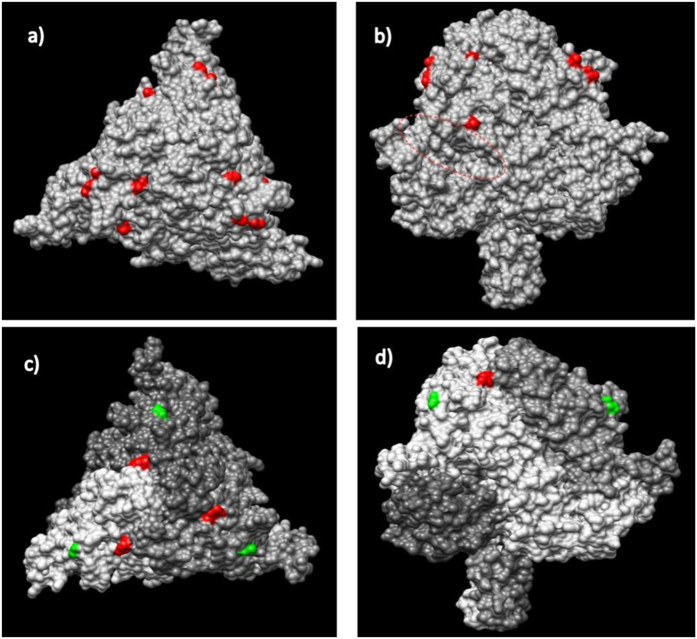
Top view (left) and lateral view (right) of the prefusion CDV F protein trimer of the viral strain detected in a *Balaenoptera physalus* predicted through homology modelling. In figure (**a** and **b**) surface aminoacids where the viral protein differed between *Stenella coeruleoalba* strain sampled during 1990–1992 epidemic and *Balaenoptera physalus* one are highlighted in red. The cleft involved in the interaction with the H protein is marked by a dotted ellipse. In figure (**c** and **d**) surface amino-acids where viral protein of *Balaenoptera physalus* strain differed from both *Stenella coerulealba* and *Globicephala melas* are highlighted in red, while those differing only from *Globicephala melas* are highligted in green.

Reliable (i.e. experimentally determined) structural templates were identified for the N, M, F and H proteins and consequently the tertiary/quaternary structure was predicted only for these proteins. Particularly, based on the available templates, the following regions of each protein were consistently modeled: N protein (aa 3–401) (Fig. 3), M protein (aa 20–326) (Supplementary Figure S1), F protein (aa 26–487) (Fig. 4) and H protein (157–603) (Supplementary Figure S2).

Non-synonymous changes compared with the AJ608288 sequence were located in the core of H (aminoacid 451) and M (aminoacids 59–191) proteins, while similar variations were expressed on the protein surface of the F protein (aminoacids 52, 151,154, and 161) (Fig. 4) or were part of its signal peptide (aminoacid 3). The only aminoacid change modeled in the N protein (aminoacid 21) was located in the inner part of the capsid that could potentially interact with viral RNA (Fig. 3). Unfortunately, aminoacidic changes located in codon 436, 462, and 509 of the same protein were located in regions that could not be reliably modeled by homology modeling. When our sequence was compared with strains HQ829973 and HQ829972, isolated during the 2006–‘08 outbreak, the following non synonymous substitutions (Table 1) were identified: HQ829972 differed in His509Arg (N protein), Ser116Gly and Ser249Gly (P/V/C protein), Ser191Leu (M protein) (Supplementary Figure S1), Thr451Ala (H protein), and Ala2Val, Ser3Ala, Ser151Asn and Arg161Gln (F protein) (Fig. 4). HQ829973 differed in Pro454Leu and His509Arg (N protein), Ser116Gly and Ser249Gly (P/V/C protein), Ser191Leu (M protein) (Supplementary Figure S1), Thr451Ala (H protein) and Ser3Ala and Arg161Gln (F protein) (Fig. 4) (Table 1).

Furthermore, analysis of sites under episodic diversifying selection revealed the presence of 6, 5, 2, 4 and 17 sites under this kind of selection respectively in the F, H, M, N and P/V/C genes of viruses affecting different host species. Remarkably, these include codons 151 of the F gene, 451 of the H gene, 59 of the M gene and 249 of the P/V/C gene that were demonstrated to vary among the strains considered in the present study.

## Discussion and Conclusions

In the present work, an in-depth investigation of the whole genome of DMV recovered from a fin whale has been carried out, in order to understand whether and how the genomic and aminoacidic changes encountered could affect the virus-host interaction dynamics and, consequently, the infection’s pathogenesis^11^. After these investigations, the obtained sequences were compared with those available from a striped dolphin and a long-finned pilot whale stranded during the 2 most relevant outbreaks in the Mediterranean Sea (1990–1992 and 2006–2008). The comparison of the genomic sequences reveled several differences between the strains, with a number of non-silent mutations being identified in all considered genes.

The 2 most relevant substitutions, Ile21Thr on the N protein and Asn52Ser on the F protein, were noted comparing the fin whale DMV isolate to those of the 1990–’92 epidemic. The 2 aforementioned variations, observed also in the DMV sequence isolated during the 2006-‘08 outbreak, are considered as relevant since they were predicted to be located on the protein surface when investigating their tertiary structure.

In this respect, the nucleoprotein constitutes the viral capsid and has a major role in the binding and protection of the viral RNA, which is embedded in a pocket located on the upper surface of the capsid. The detected mutation affects the inside-bottom of the protomer, a negatively charged region which interacts with the positively charged, outside-top one, located in the underlying layer of the nucleocapsid helix^18^. Remarkably, while Isoleucine is a hydrophobic aminoacid, Threonine is a hydrophilic one and, consequently, this substitution could have a relevant effect on the biochemical properties of that region potentially interacting with viral RNA. Nevertheless, that Ile21Thr could actually play a role in nucleocapsid assembly or RNA binding and replication remains impossible to be proven without experimental evidences.

Among the 6 non-synonymous changes observed on the F protein’s surface, the Asn52Ser substitution is located near a cavity formed by two adjacent monomers. This cleft displays several hydrophobic residues that have been reported for other morbilliviral species to be involved in the interaction with H antigen through hydrophobic protein-protein interactions^19^. Experimental substitution of hydrophobic residues with polar or charged ones through site directed mutagenesis impaired the fusion activity without relevantly affecting protein stability and structure, probably as a consequence of an impairment of the protein interacting surfaces. It has been also suggested that a decrease in the avidity of H/F interaction, after an initial increase in fusion activity, leads to a lack of appreciable binding and consequent fusion impairment^11^. In our study, the hydrophobic index changed from −3.5 (dolphin) to −0.8 (whale) according to Kyte & Doolittle (1982) scale, thus supporting the presence of a less hydrophilic region.

The 2 aforementioned relevant substitutions (Ile21Thr on the N protein and Asn52Ser on the F antigen) were already present in the viral strain isolated from striped dolphins and long-finned pilot whales during the 2006–‘08 DMV outbreak in Spanish waters. The additional mutations, occurred after the 2006–‘08 outbreak, could have favored the different DMV pathogenetic behavior in fin whale. In fact, despite morbilliviral infection had previously been described in mysticetes^3^,^4^,^20^,^21^,^22^,^23^, an epidemic cluster was recently reported in this species in the Mediterranean basin without any temporal relation with ongoing epidemic outbreaks^2^. Still worthy to be mentioned, a relevant proportion of the aminoacids which differentiate the strains sampled from the two epidemics and from the fin whale herein investigated were proven to be under diversifying selection, thus supporting their potential role in DMV host switch.

Notwithstanding the above, a number of alternative hypotheses can be drawn. The absence of DMV-infected mysticetes until 2011^2^ could be due to a reduced monitoring and/or diagnostic capability, provided that a peak in fin whale strandings was noted in 2008 (Italian Cetacean Strandings’ Database); also a reduced population’s anti-viral immunity, possibly related to environmental contaminants^24^,^25^, could be considered a further possible explanation. Furthermore, as in the case of CDV, an increased infectious pressure, together with an endemic viral circulation could have increased the possibility of a DMV jump into new hosts^9^,^26^,^27^, as exemplified by the case of infection recently described in a captive harbor seal^14^, which could be the likely results of spill-over events similar to those occurring in endemic viral circulation.

The phylogenetic tree obtained in the present study evidences the close relation between DMV strains circulating in the Mediterranean with viruses circulating in the Atlantic ocean approximatively between 2005 and 2007. Nevertheless, the viral sequence characterized in the fin whale herein reported compared with those detected in striped dolphins by Bento and coworkers^28^ between 2011 and 2014 shows a relative distance. These data thus support the idea of a possible divergence between the viral strain circulating in the Mediterranean and Southern adjacent waters of the Eastern Atlantic Ocean and those characterized from Atlantic waters of the Iberic Peninsula^28^. These observations support the suggestions from other authors of a relative isolation among these cetacean populations with the virus entering in or exiting from the Mediterranean Sea interacting with the cetaceans’ population from the Canary Islands^11^,^12^,^29^,^30^. Despite these considerations, the data herein reported regarding the apparent change DMV pathogenicity for fin whales as well as the changes in the viral sequence introduce an additional hypothesis of a constant and prolonged morbilliviral infection’s presence among Mediterranean cetaceans.

Regarding other notable variations, as with other morbilliviruses, the most relevant changes shared by the herein investigated viral sequence and those of a striped dolphin from the 1990–’92 epidemic are expected to be sited in the H gene codifying for the structural protein involved in viral attachment through interaction with the SLAM/CD150 cell receptor^2^,^11^,^15^,^31^. The phylogenetic tree of this viral gene was proven to overlie, with few exceptions, that of the host SLAM gene, arguing in favor of their co-evolution^15^,^32^. However, at the same time, few changes in the receptor binding region have been reported to affect both the viral tissue receptor and host tropism^32^. Nevertheless, the only 2 aminoacidic differences identified between the H antigen of the fin whale under study and that of the striped dolphin were located in the protein core, with no direct effect on the interaction with the host and, possibly, without playing a relevant role also in cross-species infection. Similar considerations can be drawn for the M gene, with the 2 detected substitutions being not exposed on the protein surface. Anyway, potential effects on the viral protein’s structure, processing or stability, with repercussions on cell and host tropism, cannot be excluded *a priori*. Further insight could originate from reverse genetic-based studies on protein-to-protein interaction and metabolism and from the crystallographic analysis of the tertiary/quaternary structure of DMV structural and non-structural antigens alongside viral receptor proteins, as reported in the herein investigated fin whale, in order to better understand the interaction of the “mutated” virus with host cell receptors and the role of single protein domains.

Additionally, the herein reported Ala3Ser polymorphism in the F antigen’s primary structure should be also underlined. Although this variation was located in the signal peptide, the F antigen’s interaction with the host’s cell membrane and the role of the single domains are still unclear, especially for DMV.

Finally, several synonymous substitutions were identified in the present study. Despite these mutations are often neglected, they could still have an impact on viral fitness and host tropism by modifying several features like viral protein synthesis and transcription, RNA secondary structure, and codon bias, just to mention a few. Thus, considering the results of previous studies carried out on the corresponding domains of CDV^33^, possible changes in the above described cleft region could affect the interaction dynamics between the H and F viral antigens, on one side, and the target host cells, on the other.

In conclusion, this work underscores the relevance of structural investigations, with the specific aim to gain an appropriate understanding of viral behavior. Indeed, we believe that the peculiar structural modifications of DMV herein reported may have contributed, with the likely, additional support of “complementary” structural changes affecting the SLAM/CD150 cell receptor (and, possibly, other viral receptors), for the putative shift in the pathogenicity of this viral strain towards certain species^2^,^11^,^14^,^34^. These affected cetaceans species, among which evidence of trans-placentally acquired DMV infection has been also documented^2^,^13^,^34^, could act as reservoirs for the virus, similarly to wild carnivores with CDV^9^,^27^, thereby acting as a potential source of spill-back events involving immunologically naive populations of “canonical” hosts and thus supporting DMV persistence and circulation in the Mediterranean Sea.

## Methods

### Samples

The samples used in this research project, in order to study the DMV complete viral genome, were obtained from a newborn male fin whale’s found stranded alive on Elba Island (Tuscany)^2^ and died after a few hours. Post-mortem investigations were conducted within 24 hours of death and animal tissues were sampled during on field necropsy using RNAlater stabilization Solution (Thermo Fisher Scientific).

Tissues for this project have been provided by the Mediterranean Marine Mammal Tissue Bank, Department of Comparative Biomedicine and Food Science, University of Padova, viale dell’Università 16, 35020 Legnaro - Agripolis PD, Italy

### Viral RNA extraction and retrotranscription

Total RNA extraction from brain, lung, and spleen tissue was performed using PureLink RNA Mini Kit (Ambion, Thermo Scientific) following manufacturer’s instructions. The obtained RNAs were quantified using NanoDrop 1000 (Thermo Scientific) and the corresponding cDNAs were obtained employing a previously published primer named DMV2^35^. From six to eight micrograms of total RNA were used for the retrotranscriptase reaction carried out according to manufacturer’s indications (RevertAid First Strand cDNA Synthesis Kit, Thermo Scientific).

### Primer design, PCR protocol and cloning procedures

15 pairs of primers were used to perform conventional PCR; 4 of these pairs and 2 single primers (DMV-N1 and DMV-P2, DMV-10 pair, DMV-11 pair and DMV-12pair; DMV-C and DMV-F6) have been previously used to detect different DMV fragments^2^,^35^ (Table 2). The other set of primers (Table 2) were designed using Prime3 based on the available DMV gene sequence Genbank Acc. No. AJ608288, in order to detect all the five transcription units coding the nucleocapsid protein (N), the phosphoprotein (P), the matrix protein (M), the fusion glycoprotein (F), the hemagglutinin (H) as well as two virulence factor proteins (C and V). Amplification was performed using a high-fidelity polymerase (Phusion Hot Start II DNA Polymerase, Thermo Scientific), with the following PCR conditions: 30 sec at 98 °C; 35 cycles of 10 sec at 98 °C, 30 sec at 58 °C, 1 min at 72 °C; 10 min at 72 °C. The PCR products obtained were size-separated by agarose gel electrophoresis, to be then displayed in agarose gel (3730xl DNA Analyzer, Thermo Scientific).

The PCR products obtained from lung and cerebral cDNA were purified, cloned into plasmid vector PCR-Blunt II TOPO (Thermo Scientific) according to the manufacturer’s instructions, and then sequenced. Three sequences from lung and brain plasmidic colonies were analyzed. Programs in the DNASTAR Lasergene software package (http://www.dnastar.com/t-dnastar-lasergene.aspx) were used to edit, assemble, and translate sequences.

### Primary and secondary structure prediction

In the present study, we analyzed the primary structures of N, M, F, H and P/V/C of DMV gene-encoded proteins. In order to identify differences in secondary structures between the reported DMV gene sequence and ours, each protein sequence was subjected to consensus secondary structure prediction using PSIPRED^36^.

### Nucleotide and aminoacid sequence comparison and phylogenetic analysis

Based upon their coding nature, sequences of DMV genes (i.e. H, F, M, and N genes) obtained from fin whale (present study), striped dolphin (Genbank Acc. No. AJ608288 and HQ829973), and long-finned pilot whale (Genbank Acc. No. HQ829972) were aligned at aminoacid level using the MAFFT algorithm implemented in TransaltorX and then back-translated to nucleotide. Differences between the strains as well as their effects (i.e. synonymous *versus* non-synonymous mutations) were recorded.

The relationship with other DMV and PWMV isolates was estimated performing a phylogenic analysis based on a selection of publicly available P genes.

Briefly, all sequences were aligned as previously described and a phylogenetic tree was reconstructed using the maximum likelihood method implemented in PHYML^37^. Substitution model was selected according to Bayesian Information Criterion (BIC), calculated using Jmodeltest 2.1.2^38^. The phylogenetic tree reliability was evaluated using the Shimodaira–Hasegawa [SH]-aLRT^39^ likelihood-based measures of branch supports implemented in PhyML.

### Homology modelling

In order to evaluate the morphological changes caused by non-synonymous substitutions, the tertiary and/or quaternary structure of relevant proteins were evaluated and compared between strains collected from different hosts.

*S*equences of DMV genes (i.e. H, F, M and N genes) collected from fin whale (present study), from striped dolphin (Genbank Acc. No. AJ608288 and HQ829973), and from long-finned pilot whale (Genbank Acc. No. HQ829972) were translated at aminoacid level and the best protein template for which tertiary or quaternary structure had been experimentally determined was searched using the SWISS-MODEL web server^40^. Those sequences were selected because they represent the oldest sequences available in Genbank (i.e. Genbank Acc.No. AJ608288) and phylogenetically highly related strains obtained from hosts different from fin whale (i.e. Genbank Acc. No. HQ829972 and HQ829973).

The same program was used for modeling the protein structure through an homology modeling approach^41^. For each protein the structural alignment was performed using the MatchMaker method implemented in Chimera^42^. This approach performs a fit after automatically identifying which residues should be paired using both primary and secondary structure, allowing similar structures to be superimposed even when their sequence similarity is low.

### Diversifying selection

In order to identify aminoacid codons potentially involved in host adaptation, we tried to detect sites of different proteins characterized by a frequency of non-synonymous substitutions among different hosts that was greater in comparison with that expected by chance. A collection of representative complete genomes (Genbank Acc. No: AY443350, AB490679, KF856711, KM926612, EU726268, AB687720, KP793921, JX681125, KP677502, HM852904, AB924122, AB012948, KP868655, KT633939, JX217850, KJ867545, NC_028249, AB547189, NC_005283, KR337460 and AJ608288) of *Morbillivirus* affecting different host species was downloaded from Genbank and each protein was identified, aligned as previously described and analyzed separately. The analysis of both pervasive and episodic selection was performed using the MEME method^43^ implemented in HyPhy^44^. The level of significance was set to p-value < 0.05.

## Additional Information

**How to cite this article**: Beffagna, G. *et al*. Genomic and structural investigation on dolphin morbillivirus (DMV) in Mediterranean fin whales (*Balaenoptera physalus*). *Sci. Rep.* **7**, 41554; doi: 10.1038/srep41554 (2017).

**Publisher's note:** Springer Nature remains neutral with regard to jurisdictional claims in published maps and institutional affiliations.

## Supplementary Material

Supplementary Information

## Figures and Tables

**Table 1:** Non-synonymous aminoacid variations in each DMV single gene between fin whale (*Balaenoptera physalus*), striped dolphin (*Stenella coeruleoalba*) (Genbank Acc. No. AJ608288 and HQ829973) and long-finned pilot whale (*Globicephala melas*) (Genbank Acc. No. HQ829972) strains*.

AA position	AA in AJ608288	AA in HQ829973	AA in HQ829972	AA in fin whale
**N GENE**				
21	Ile	Thr	Thr	Thr
436	Ala	Thr	Thr	Thr
454	Pro	Leu	Pro	Pro
462	Thr	Ala	Ala	Ala
509	Arg	Arg	Arg	His
**P/V/C GENE**				
116	Gly	Gly	Gly	Ser
119	Ala	Glu	Glu	Glu
163	Asn	Ser	Ser	Ser
249	Gly	Gly	Gly	Ser
313	Met	Leu	Leu	Leu
**M GENE**				
59	Leu	Val	Val	Val
191	Leu	Leu	Leu	Ser
**F GENE**				
2	Ala	Ala	Val	Ala
3	Ala	Ala	Ala	Ser
52	Asn	Ser	Ser	Ser
151	Asn	Ser	Asn	Ser
154	Thr	Ala	Ala	Ala
161	Arg	Gln	Gln	Arg
162	Gln	Arg	Arg	Arg
**H GENE**				
164	Val	Ala	Ala	Ala
451	Ala	Ala	Ala	Thr

**Table 2:** RT-PCR primer sets*.

Primer name	*nt* position (referred to AJ608288)	5′ → 3′ sequence (sense)	Fragment length, bp
DMV-2^35^	15702–15684	ATHCCCAGCTTTGTCTGGT	cDNA production
DMV-1F	72–92	TCAATTGGCACAGGATTTGG	474
DMV-1R	545–525	CCAATGGGTTCCTCTGGTGT	
DMV-2F	501–521	TCTATTCAAGCAGGGGAGGA	622
DMV-2R	1122–1102	TCGGCTGTGATCCCTAGTTC	
DMV-N1^35^	1203–1222	CAAGAGATGGTCAGGAGATC	1358
DMV-P2^35^	2541–2521	GACAGGTGGTGCAACCCGAC	
DMV-C^35^	2132–2152	ATGTTTATGATCACGGCGGT	769
DMV-4R	2900–2880	AGGTGGCCTTCGATAGTTGA	
DMV-5F	2439–2459	ACCAATTCCAACCTCAGTGC	716
DMV-5R	3154–3134	ATCCCACAGCAGAGCTCATT	
DMV-6F	5178–5198	TGGTCGTCAACATTGAGTCAC	690
DMV-F6^35^	5852–5835	CGCAAGACAGCTGGTGC	
DMV-7F	5684–5704	GCCCTTCATCAGTCCATCAT	667
DMV-7R	6334–6314	ATTGTTGGAGCAACGGACTC	
DMV-8F	6147–6167	CAGAGGTCAAGGGGGTGATA	700
DMV-8R	6827–6807	CGACAGTGCCTCCTACAACA	
DMV-9F	6482–6502	GGCACCATAATTAGCCAGGA	778
DMV-9R	7240–7220	CCTGCAATGGCAAGTAGTCC	
DMV-10F^2^	7206–7226	GGGTGTGCTAGCCGTTATGT	718
DMV-10R^2^	7904–7884	TTCGTCCTCATCAATCACCA	
DMV-11F^2^	7799–7819	CCGAACCTGATGATCCATTT	612
DMV-11R^2^	8411–8391	CGTAAATGTCCATCCCTGCT	
DMV-12F^2^	8290–8310	AACCGGATCCCAGCTTATG	800
DVM-12R^2^	9070–9050	CCAGGTGCACTTCAGGGTAT	
DMV-14F	3037–3057	CCAGCAGTCGAGAGAAATCC	723
DMV-14R	3759–3739	TCTCATTTAACCCCGCTGTC	
DMV-15R	3464–3484	CTGGGATGTCAAGGGGTCTA	612
DMV-15F	4075–4055	GCCTGTGGGTCTCTCATCAT	
DMV-16R	3920–3942	CAGACTCTCAGACAATGGATGC	586
DMV16F	4505–4485	GCTCTGTTGATTCTGCTGGA	

^*^nt = nucleotide; bp = base pair.
