# Supplementary material for: Genomic and structural investigation on dolphin morbillivirus (DMV) in Mediterranean fin whales (Balaenoptera physalus)
Source: Sci Rep. 2017 Jan 30;7:41554. doi: 10.1038/srep41554 (PMC5278511; doi:10.1038/srep41554)

Genomic and structural investigation on dolphin morbillivirus (DMV) in Mediterranean fin whales (*Balaenoptera physalus*)

Giorgia Beffagna[1,§], Cinzia Centelleghe[1,§,*], Giovanni Franzo[2], Giovanni Di Guardo[3], and Sandro Mazzariol[1]

[1]: *Department of Comparative Biomedicine and Food Science, University of Padua, Padua, Italy*

[2]: *Department of Animal Medicine, Production and Health, University of Padua, Padua, Italy*

[3]: *University of Teramo, Faculty of Veterinary Medicine, Teramo, Italy*

§: These authors equally contributed to this article.

Supplementary Figure S1. Reconstruction of the M protein (front view) of the strain detected in *Balaenoptera physalus*. The surface of the protein is depicted as a mesh to display the aminoacids (highlighted in red) where viral protein differed between strains detected in 1990-‘92 epidemic (*Stenella coeruleoalba* - violet mesh) or 2006-‘08 epidemic (*Stenella coeruleoalba* and *Globicephala melas* – blue mesh) and *Balaenoptera physalus* one.

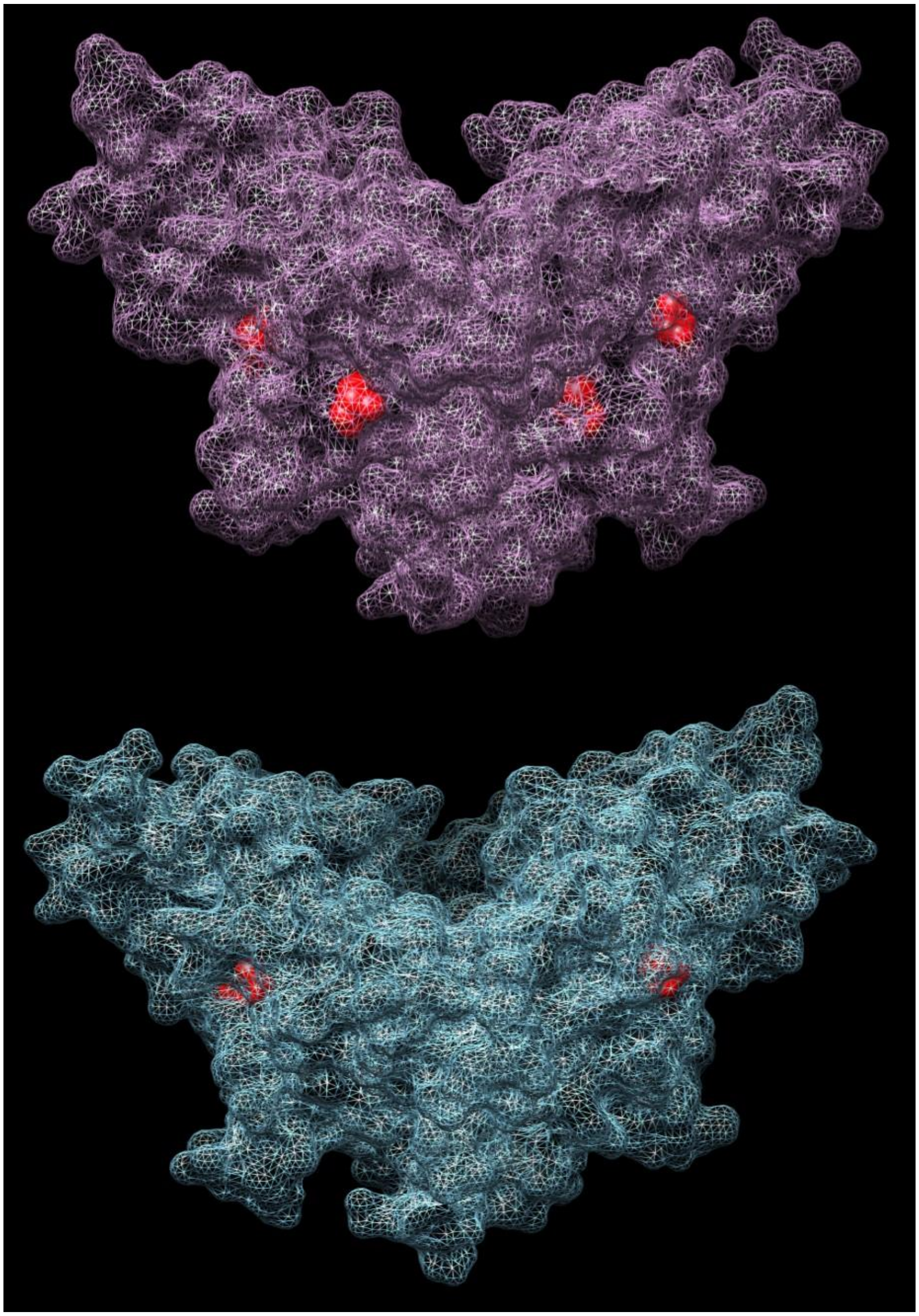

Supplementary Figure S2. Reconstruction of the H protein of the strain detected in *Balaenoptera physalus* (grey) bounded to the SLAM/CD150 receptor (light brown). The binding region between has been H protein and SLAM/CD150 receptor has been predicted based on Crystal structure of the measles virus hemagglutinin bound to its cellular receptor SLAM/CD150 (PDB:3ALZ). The surface of

the protein is depicted as a mesh to display the aminoacids (highlighted in red) where viral protein differed between *Stenella coeruleoalba* related strain and *Balaenoptera physalus*.

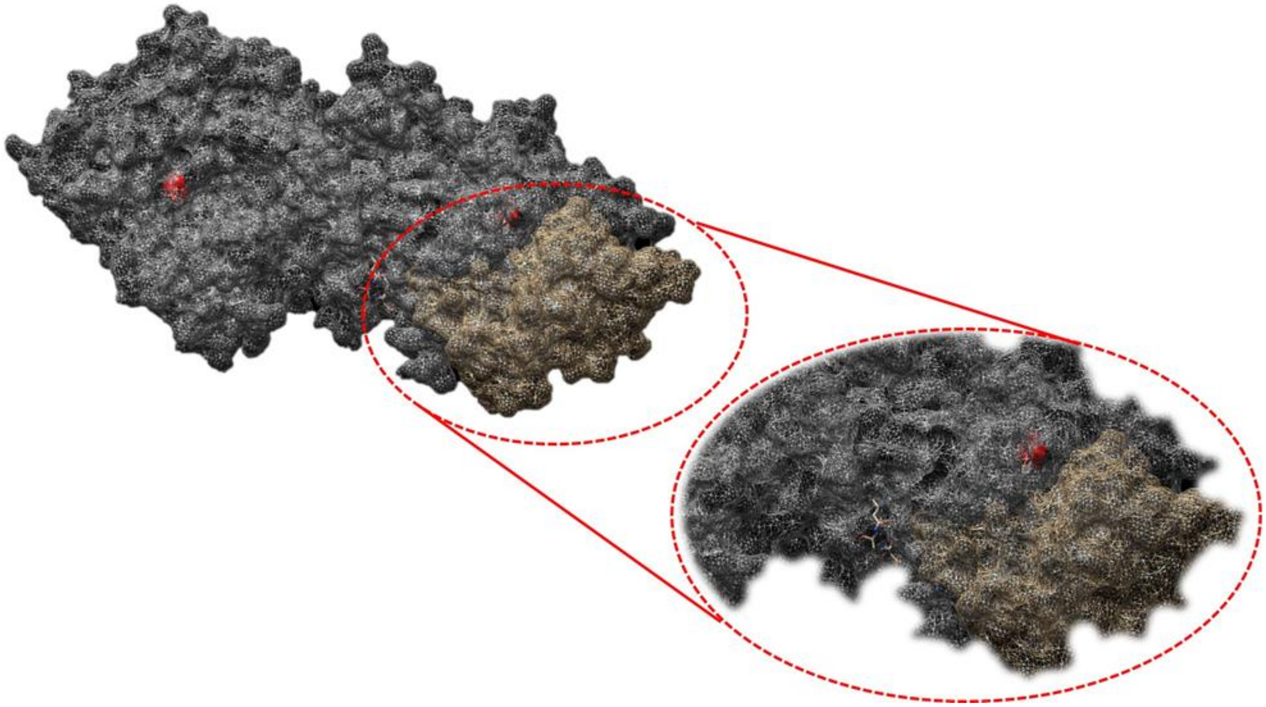

Supplement: Supplementary Information [file srep41554-s1.pdf]
